# Editorial: Characteristic clinical immune phenotypes and molecular mechanisms associated with inflammatory diseases

**DOI:** 10.3389/fimmu.2024.1384971

**Published:** 2024-03-11

**Authors:** Shuming Pan, Di Xie, Chengjin Gao, Kun Xiong

**Affiliations:** ^1^ Emergency Department, Xinhua Hospital Affiliated to Shanghai Jiao Tong University School of Medicine, Shanghai, China; ^2^ Department of Anatomy and Neurobiology, School of Basic Medical Science, Central South University, Changsha, Hunan, China

**Keywords:** immune phenotypes, molecular mechanisms, inflammatory diseases, sepsis, innate immune, adaptive immune

The whole-body inflammatory response is evident in inflammatory diseases, where the immune system secretes many related cytokines, leading to a powerful immune response. Both innate and adaptive immune cells are involved, causing local organ inflammation, including neuroinflammation, and inflammation in distant target organs.

Gut-microbiota-brain axis (GMBA) is a potential treatment to decrease the risk of nervous system impairment following TBI. The GMBA is a complex circuit that operates through the crosstalk of the gut microbiome, enteric nervous system, Chen et al. found that the gut microbiota of Phosphoglycerate mutase 5 (Pgam5)−/− mice partially relieved the lack of Pgam5 in the initial inflammatory factors and improvement of motor function after TBI following antibiotic treatment. Knocking out the Pgam5 gene led to an increase in the abundance of A. muciniphila in mice. The increasing life expectancy has led to a higher incidence of age-related neurodegenerative conditions. Within this framework, neuroinflammation emerges as a significant contributing factor. The coexistence of coronary artery disease (CAD) and cognitive impairment is a prevalent clinical phenomenon. Despite this, there is a lack of extensive research concerning the etiology of this disease combination, the exploration of potential biomarkers, and the precise identification of intervention targets. Xu et al. demonstrated specific characteristics of gut microbiota and blood metabolites linked to the simultaneous presence of CAD and cognitive impairment. These findings offer valuable insights for identifying biomarkers associated with the CAD and cognitive impairment combination and pinpointing intervention targets to address clinical challenges.

Laurindo et al., have found that interventions focused on interleukin-driven immunomodulation, chemokine (CXC) receptors signaling and expression, cold exposure, and fibrin-targeting strategies show promising potential in reducing neuroinflammatory processes in a range of models that include conditions like Alzheimer’s disease.

Sepsis is a condition characterized by a very high mortality rate among critically ill patients, primarily due to excessive systemic inflammation that leads to the uncontrolled release of inflammatory mediators. While various studies have pointed towards the significant role of PANoptosis in tumor initiation and progression, its involvement in sepsis remains incompletely understood. Dai et al. utilized single-cell sequencing analysis to identify six distinct cell types, with a notable clustering of high PANscore in B cells and low PANscore in CD16+ and CD14+ monocytes, as well as megakaryocyte progenitors. Kong et al. CD177 and MMP8 were identified as hub genes, which were of considerable value in the early diagnosis of septic shock patients. These preliminary findings are of great significance for studying immune cell infiltration in the pathogenesis of septic shock, which should be further validated in clinical studies and basic studies.

The discovery of novel clinical markers enhances our understanding of the pathogenesis of asthma. Considering the significant role that lymphotoxin-related inducible ligand (LIGHT) plays in asthma, it has the potential to be a target for asthma treatment. The aim of this study was to evaluate whether circulating microRNAs (miRNAs) targeting LIGHT could serve as diagnostic biomarkers for distinguishing asthma. Hu et al. found that plasma miR-140-5p and miR-107 can be utilized as diagnostic biomarkers to differentiate between patients with asthma and healthy controls. These miRNAs may also play a role in asthma pathogenesis by negatively regulating LIGHT.

Diabetic retinopathy (DR) is a primary cause of vision impairment globally. Recent research has emphasized the significant influence of circadian rhythms (CR) on normal retinal function in response to external light stimuli. Nevertheless, the involvement of circadian rhythms in the pathogenesis of DR and the evaluation of potential therapeutic compounds are still ambiguous. Ling et al. findings identified key genes associated with circadian rhythms and relevant drugs in diabetic retinopathy (DR), offering a new perspective on the mechanisms of DR and potential implications for future treatments. This study enhances our comprehension of circadian rhythms in DR and their significance for upcoming therapeutic approaches. Differentiating between acute respiratory distress syndrome (ARDS) phenotypes is crucial for tailored treatment. This study aimed to identify ARDS phenotypes using metabolic and autophagy-related genes in conjunction with infiltrated immune cells. Xia et al. pinpointed hub genes associated with autophagy and metabolism in ARDS and categorized patients into distinct molecular and immunophenotypes. Their findings offer valuable insights for advancing precision medicine in ARDS patient management. Xu et al. conducted a comprehensive review exploring the immunological mechanisms underlying sepsis-induced ARDS, the heterogeneity of ARDS, and current research on targeted treatments. The aim of the review is to enhance understanding of the mechanisms involved and provide insights for the precise treatment of ARDS or sepsis-ARDS. AKT serine/threonine kinase 3 (AKT3) is implicated in the pathogenesis of lung cancer, yet its involvement in ventilator-associated pneumonia (VAP) remains uncertain. Zhu et al. propose the necessity for additional verification of AKT3’s immunoregulatory role in lung cancer. Furthermore, the depletion of macrophages alleviates lung damage through the modulation of the AKT3/Glutathione Peroxidase 4 (GPX4) pathway during VAP. A bidirectional relationship exists between obesity and depression. Ninla-aesong et al. examined whether the concurrent presence of obesity and depression heightens the likelihood of severe depression and a heightened risk of suicide in adolescents diagnosed with major depressive disorder (MDD). Furthermore, they delved into the potential underlying mechanisms that connect the co-occurrence of obesity and depression to adverse outcomes in these individuals. Keratoconus (KC) is an ocular disease characterized by bilateral progressive corneal ectasia, arising from intrinsic biomechanical instability. Niu et al. discovered clusters of corneal stromal cells and immune cells that likely contribute significantly to the progression of KC by modulating immunological aspects and preserving cellular stability.

Neuroinflammation is a common feature of many neurological diseases, and remains crucial for disease progression and prognosis. Wang et al.’s study demonstrated that the long non-coding RNA Xist can modulate the activation of microglia and astrocytes in the periventricular white matter of rats with cecal ligation and puncture-induced sepsis. This regulation occurs through the miR-122-5p/PKCη axis, consequently contributing to sepsis-associated neuroinflammation. Tourette syndrome (TS) has been linked to immunological dysfunction, with the dopamine (DA) system playing a significant role in the development of TS symptoms such as behavioral stereotypes. Previous research has hinted at the presence of hyperpolarized M1 microglia in the brains of individuals with TS. However, the precise involvement of microglia in TS and their interactions with dopaminergic neurons remain uncertain. A study by Wang et al. revealed that activated microglia exhibit M1 polarization, leading to the transmission of inflammatory damage to striatal dopaminergic neurons and disruption of normal dopamine signaling.

Cell death is an essential process in organisms. Different forms of cell death can be classified into controlled and uncontrolled types, such as apoptosis, necroptosis, and ferroptosis. Ferroptosis is an iron-dependent and non-apoptotic form of cell death caused by oxidative-reduction imbalance. ferroptosis primarily occurs through transporter-dependent and enzyme-regulated pathways. Wang et al. found that in the transient middle cerebral artery occlusion (tMCAO) mouse model, a reduction in Treg cells protected against the activation of astrocytes and significantly reduced the expression levels of

Ferroptosis suppressor protein-1(FSP1), Interleukin-6 (IL-6), IL-2, and NOD-like receptor pyrin domain-containing protein 3 (NLRP3) while partially reversing the changes in Treg cells. Mechanistically, the reduction of Treg cells alleviated renal fibrosis post-cerebral infarction by regulating IL-10/GPX4. Treg cells play a crucial role in the development of secondary injuries in the disease. Increasing evidence suggests a link between ischemic stroke and Treg, although its underlying mechanism is unclear.

Macrophages are important immune cells in the innate immune system, with significant diversity and polarization. Under pathological conditions, in addition to resident macrophages, other macrophages are recruited to the diseased tissue and polarized into various phenotypes, regulating the function of target organs. Ma et al. found that the knockout of the angiopoietin-like 3 (Angptl3) gene significantly alleviated renal dysfunction and epithelial-mesenchymal transition in diabetic nephropathy mice. Both *in vivo* and *in vitro* studies showed that the knockout of the Angptl3 gene promoted the transition of pro-inflammatory macrophages to anti-inflammatory macrophages, improved the downregulation of glomerular proteinuria, synaptopodin, and intrapodatin, inhibited the activation of NLRP3 inflammasomes and the release of IL-1β, and regulated the expression of α-SMA through macrophage polarization. Integrin αLβ2 (also known as CD11a/CD18 or CD11a) is a significant leukocyte adhesion molecule crucial for leukocyte arrest and the formation of immunological synapses. Despite its established role in these processes, its significance in bone marrow remains underexplored. In this study, we demonstrate the ubiquitous expression of CD11a across all subsets of hematopoietic stem and progenitor cells (HSPCs). Concurrently, a study by Hou et al. revealed a substantial upregulation of IL-27 production, a key cytokine responsible for promoting HSPC proliferation, both *in vivo* and *in vitro*. This finding unveils a new dimension to the role of CD11a in cellular biology. The progression of osteoarthritis (OA) involves various factors, with cartilage erosion as the fundamental pathological mechanism of degeneration, closely associated with chondrocyte apoptosis. Research by Yu et al. indicated that apoptosis-related genes (ARGs) have the potential to foresee the onset of OA and could be linked to various stages of OA advancement. Myopia is the most common type of refractive error and has emerged as one of the main reasons for visual impairment. With the rising rate of myopia, there is a growing necessity to comprehend the factors responsible for its occurrence. Inflammation, a fundamental physiological process in humans, is reviewed by Xu et al. to thoroughly explain the connection between myopia and inflammation. Low-grade inflammation in the body can prompt the progression of myopia, and it has been observed that the prevalence of myopia is higher in individuals with inflammatory or immune disorders. Takotsubo syndrome (TTS) is a condition marked by temporary cardiac dysfunction exhibiting regional wall motion abnormalities in the ventricles, believed to stem mainly from the impact of a rapid surge in catecholamines on the heart. Accumulating findings from both clinical and fundamental research studies consistently underscore the link between inflammation and the development, advancement, and resolution of TTS (Lim et al.). Wound repair presents a multifaceted challenge for both medical professionals and researchers. Traditional methods of wound healing have shown various constraints such as extended treatment periods, elevated costs, and imposing economic and emotional burdens on patients. According to Qin et al., Mesenchymal Stem Cell Exosomes (MSC-Exos) prove to be more effective than alternative wound therapies. In essence, although exosomes derived from different MSC sources possess unique differentiation capacities, they demonstrate enhanced consistency in promoting wound healing.

Due to the different academic backgrounds of the researchers, each of the above studies can be considered as an independent research field. However, these studies, as a whole, provide insights into the impact of immune cell-mediated inflammatory responses on organ function. Therefore, considering the trend and urgency in this field, we believe that this topic will attract the attention of researchers in related fields, including drug developers and clinical scientists. This Research Topic will also provide some cutting-edge insights into the development of this field, particularly in research related to the gut-brain axis. This Research Topic is dedicated to promoting the publication of original findings and providing novel insights.

## Author contributions

SP: Supervision, Writing – original draft. DX: Writing – original draft. CG: Conceptualization, Supervision, Writing – review & editing. KX: Validation, Writing – review & editing.

